# Vision and Visual History in Elite/Near-Elite-Level Cricketers and Rugby-League Players

**DOI:** 10.1186/s40798-017-0106-z

**Published:** 2017-11-10

**Authors:** Brendan T. Barrett, Jonathan C. Flavell, Simon J. Bennett, Alice G. Cruickshank, Alex Mankowska, Julie M. Harris, John G. Buckley

**Affiliations:** 1 School of Optometry and Vision Science, University of Bradford, Bradford, UK; 2 Department of Psychology, University of York, York, UK; 3 School of Sport and Exercise Science, Liverpool John Moores University, Liverpool, UK; 4 School of Psychology and Neuroscience, University of St Andrews, St Andrews, UK; 5 School of Engineering, University of Bradford, Bradford, UK

**Keywords:** Vision, Eye, Eyesight, Elite sports, Ametropia, Refractive error, Cricket, Rugby league

## Abstract

**Background:**

The importance of optimal and/or superior vision for participation in high-level sports remains the subject of considerable clinical research interest. Here, we examine the vision and visual history of elite/near-elite cricketers and rugby-league players.

**Methods:**

Stereoacuity (TNO), colour vision, and distance (with/without pinhole) and near visual acuity (VA) were measured in two cricket squads (elite/international-level, female, *n* = 16; near-elite, male, *n* = 23) and one professional rugby-league squad (male, *n* = 20). Refractive error was determined, and details of any correction worn and visual history were recorded.

**Results:**

Overall, 63% had their last eye examination within 2 years. However, some had not had an eye examination for 5 years or had never had one (near-elite cricketers 30%; rugby-league players 15%; elite cricketers 6%). Comparing our results for all participants to published data for young, optimally corrected, non-sporting adults, distance VA was ~ 1 line of letters worse than expected. Adopting *α* = 0.01, the deficit in distance VA was significant, but only for elite cricketers (*p* < 0.001) (near-elite cricketers, *p* = 0.02; rugby-league players, *p* = 0.03). Near VA did not differ between subgroups or relative to published norms for young adults (*p* > 0.02 for all comparisons). On average, near stereoacuity was *better* than in young adults, but only in elite cricketers (*p* < 0.001; *p* = 0.03, near-elite cricketers; *p* = 0.47, rugby-league players). On-field visual issues were present in 27% of participants and mostly (in 75% of cases) comprised uncorrected ametropia. Some cricketers (near-elite 17.4%; elite 38%) wore refractive correction during play, but no rugby-league player did. Some individuals with prescribed correction choose not to wear it when playing.

**Conclusions:**

Aside from near stereoacuity in elite cricketers, the basic visual abilities we measured were not better than equivalent, published data for optimally corrected adults; 20–25% exhibited sub-optimal vision, suggesting that the clearest possible vision might not be critical for participation at the highest levels in the sports of cricket or rugby league. Although vision could be improved in a sizeable proportion of our sample, the impact of correcting these, mostly subtle, refractive anomalies on playing performance is unknown.

**Electronic supplementary material:**

The online version of this article (doi:10.1186/s40798-017-0106-z) contains supplementary material, which is available to authorized users.

## Key Points

- Around two thirds of elite- and near-elite-level rugby-league players and cricketers had their eyes examined in the past 2 years, but 20–25% had either never had an eye examination or had had their last examination ≥ 5 years ago.
- Twenty to twenty-five per cent had an anomaly of vision in their habitual playing state, which, in ~ 75% of cases, was due to uncorrected refractive error; such errors are easily treatable using contact lenses.
- Findings suggest that the basic, clinically measured vision of high-level cricket and rugby-league players is frequently sub-optimal. However, the extent to which correction of these, mostly subtle, anomalies of refraction would lead to improved, on-field performance is not known.

## Background

Vision plays a key role in interceptive tasks that are ubiquitous features of human action and interaction in our world, for example when shaking hands or crossing the street. Interceptive tasks are also a key component of many sports, such as when catching and/or striking a ball.

While some minimum level of vision is obviously important for participation in most sports, the requirement to optimise retinal image clarity in order to maximise sporting performance is contested. There are claims that vision is superior in elite athletes compared to the general population [1–6] or in elites compared to sub-elite athletes or novices [7–9]. Most of these claims have been made for sports which feature a small, fast moving target such as in baseball [5]. Also, vision measures and oculomotor behaviour (e.g. where and/or when the eyes are looking) may differ between elite individuals from different sports [10, 11] or between players in different positions in the same sports (e.g. fielders and pitchers in baseball [12], hitters and pitchers in baseball [13]; but see [14]). One interpretation of these findings is that excellent vision has contributed to the potential for ‘eliteness’. This is supported by studies suggesting (i) that vision can be trained (e.g. in terms of where and/or when to look) or possibly even improved (i.e. made more acute) [2, 14–17], (ii) that better vision is associated with better on-field performance [18, 19], and (iii) that vision training can enhance performance in the field [16, 20].

However, counter evidence suggests that optimal and/or superior vision is not required to fulfil the potential for eliteness. Firstly, there are examples of elite-sporting individuals whose vision was sub-optimal. Mansoor Ali Khan (1941–2011) became the captain of the Indian cricket team at the age of 21 having lost sight in one eye when aged 16 [21], and there are also questions about the vision of the legendary Babe Ruth [22]. Secondly, low to moderate levels of retinal-image blur, simulating uncorrected myopia, may not necessarily impact negatively on performance in sporting tasks [23, 24], even in tasks where the visual demands are high [25–27]. Also, the uptake of eye care amongst elite-level athletes may be low; hence, suboptimal vision (e.g. due to uncorrected refractive error) may well exist amongst some elite sportspeople [28–32]. Finally, a number of studies have concluded that claims that vision can be trained or improved remain unproven [18, 33–35].

As outlined above, the evidence that high-level sports players need to have superior vision is equivocal. The importance of ‘vision’ depends not only on the sport, and in some cases, on the position played in that sport (e.g. bowling or batting or fielding in cricket), but also on precisely which aspect of *vision* is being considered (e.g. which of the many different tests of vision are used to evaluate vision). While visual acuity (the ability to resolve static, black letters of decreasing size on a white background) is the most well-known measure of acuteness of vision, there are many measures that reflect different visual abilities; these include stereoacuity, visual acuity for dynamic targets (‘dynamic VA’) [4, 12, 36], contrast sensitivity [37], and positional acuity [38], to name but a few. Different measures of vision may reflect, to a greater or lesser extent, the demands associated with particular tasks on the field of play. Further, if they exist at all, differences in vision between elites and sub-elites or novices may not emerge as differences in raw visual performance measures, but they might instead arise from differences in *how* vision is used. That is, elites, either through experience or through training, may adopt a better strategy than sub-elites for knowing where and when to look in order to access the most useful information at the most appropriate time [39]. For example, in a study of anticipation and visual search behaviour by Savelsbergh et al. [40] in which expert soccer goalkeepers were classified as successful or unsuccessful based on performance on a film-based test of anticipation, the ‘successful’ experts appeared to spend longer periods of time fixating on the non-kicking leg compared with non-successful experts. In putting, Vickers [41] found that better golfers exhibited longer fixation durations on the ball and target and fewer fixations on the club and surface. In cricket, Land and McLeod’s study [42] led them to conclude that a cricket player’s eye movement strategy contributes to skill in the game (see also [43]).The distinction between visual ability measures and the use of different visual strategies has been compared to the hardware versus software (respectively) distinction in computing [44]. Adopting this analogy, many believe that while changes to the hardware (improvements in visual ability, e.g. visual acuity and stereoacuity) are not achievable (but see [20]), changes to the software (e.g. improvements in visual strategies) may be both possible and effective [18]. It is possible that either ‘hardware’ or ‘software’ differences in vision contribute to the potential for sporting eliteness or that neither do. It is also possible that the two might interact so that for example, reduced vision (e.g. due to significant uncorrected refractive error) may limit a player’s ability to employ a particular visual strategy.

Based upon the computing analogy outlined above, investigations into whether differences exist in vision between elites and sub-elite or novices, it is possible to divide such studies into comparisons between players and non-players in terms of vision abilities or of visual strategy. The current study is concerned with the former. Surprisingly, there have been no studies of eye care and basic (i.e. standard) visual abilities amongst UK-based high-level sportspeople. Here, we examine vision and visual history in UK-based elite/sub-elite players from two sports with very different visual demands, cricket and rugby league. The impact of sub-optimal vision may be greater in cricketers due to the demands of the game which features a small, often very fast moving object (the cricket ball). We gathered information about the visual history of our sports players to examine basic visual abilities in high-level cricketers and rugby-league players and to compare these visual measures with published normative values from young adults. Our aim was to understand the importance of optimally corrected vision for high-level participation in these sports and to look for evidence for better-than-normal vision. To our knowledge, this is the first study to measure the basic vision abilities and visual history of athletes who play the popular UK sports of cricket and rugby league.

## Methods

### Participants

Between September 2014 and October 2015, we conducted clinical visual assessments in 59 high-level sports players. Our rugby-league player sample consisted of 20 males from a ‘Super-League’ (professional) team. There were two cricketing samples. The first consisted of 23 male, near-elite-level players who represent the best players from universities in the north of England and who formed the Leeds/Bradford Marylebone Cricket Club. Several of these cricketers had played for periods with English county teams and together this group had played as a team against first-class, English county cricket teams. The second sample of cricketers was female and consisted of 16 members of England’s international women’s cricket team. Age details are provided in Table 1.

**Table 1:** Details of clinical visual measures

Group	Age (years, mean ± SD, range)	Distance visual acuity (habitual^a^) (LogMAR) (average ± SD) [range]	Number (%) showing improvement of ≥ 1 line with pinhole disc in at least 1 eye	Near visual acuity (habitual^a^) (LogMAR) (average ± SD) [range]	Stereoacuity (″, seconds of arc)	Number (%) with red/green colour vision deficit
Elite cricketers (*n* = 16)	25.6 ± 2.9 [range 22–30]	− 0.07 ± 0.08 [+ 0.10 to − 0.18] *(p < 0.001)* ^b^	1 (6%)	− 0.19 ± 0.05 [− 0.09 to − 0.26] (*p* = 0.02)^b^	Average 34.2″ Median 30″ Range 15–60″ IQR 22.5″–45″ *(p < 0.001)* ^c^	0 (0%)
Near-Elite cricketers (*n* = 23)	20.7 ± 1.5 [range 17–23]	− 0.04 ± 0.21 [+ 0.20 to − 0.18] (*p* = 0.02)^b^	2 (8.7%)	− 0.18 ± 0.22 [+ 0.77 to − 0.30] (*p* = 0.64)^b^	Average 36″ Median 22.5″ Range 15–120″ IQR 15″-52.5″ (*p* = 0.03)^c^	2 (8.7%)
Rugby players (*n* = 20)	22.8 ± 3.5 [range 17–28]	− 0.09 ± 0.13 [+ 0.24 to − 0.20] (*p* = 0.03)^b^	2 (10%)	− 0.19 ± 0.08 [0 to − 0.30] (*p* = 0.07)^b^	Average 61″ Median 30″ Range 15–240″ IQR 30″–60″ (*p* = 0.47)^c^	0 (0%)

*P* values in italics met the criterion for statistical significance (*p* < 0.01)

^a^Habitual means with both eyes open, wearing any correction, that is normally worn when playing or without correction if not

^b^Results of statistical comparison using *t* tests of distance and near (VA) visual acuity for our three samples to the average value (− 0.16 logMAR) expected in young adults with normal or correct-to-normal vision [49, 50]

^c^Results of statistical comparison using *z* testing of TNO stereoacuity for our three samples against an average value for stereoacuity measured in young adults with the TNO test (50″)[51–55]

### Protocols and Clinical Data Gathered

We gathered data from the participant groups described above. The results from our participants were compared with published data from young non-sporting individuals (see the ‘Statistical Analysis *’* section below).

We measured the monocular (each eye) and binocular visual acuities (VA) at distance (6 m) and near (40 cm). Vision was measured in the habitual ‘sports participation’ state, i.e. with optical correction if worn when playing sports. Distance vison measures were taken using a logMAR chart [45, 46], and near vision was measured using an MNRead chart [47], which has a similar scoring system to logMAR distance VA measurement. We also determined whether distance VA in each eye improved when participants viewed through a pinhole (1 mm) because any improvement would suggest that the existing habitual refractive status was non-optimal [48]. We assessed stereoacuity using the TNO stereotest (version no. 14, 2014 TNO Stereotest. Boca Raton, FL: Richmond Products). When only one of the two plates at the next level was correctly identified, stereoacuity (in seconds of arc, ″) was taken as the average of the two levels. Colour vision was assessed using the Ishihara test (24-plate edition, Kanehara & Co. Ltd., Tokyo, Japan), and we determined the type and extent of any refractive error using an auto-refractor (Shin-Nippon, NVision-K 5001, Shin-Nippon Corporation, Japan).

We recorded the frequency of eye examinations, whether glasses or contact lenses are worn while playing, and whether there was any history of eye injury or eye disease. Using a questionnaire, participants were asked about the use of an eye patch as a child and any previous participation in eye/vision-training programmes. The complete list of questions is given in Table 2. Our aim was to gather detailed information about the clinically measured visual function, the perceived level of vision, and the visual history of each participant. All but two questionnaires (both from the rugby-league sample) were completed, though not all questions were answered by every participant (Table 2).

**Table 2:** Eye examination history, refractive correction worn (including when playing), and refractive/other visual conditions identified

Group	Last eye examination			Wears specs or CLs?	Wears specs or CLs while playing?	Refractive error corrected by specs or CLs	Number (%) where residual visual issues were identified	Details of residual issues identified
	Within 2 years	2–5 years	5+ years or never					
Elite cricketers (*n* = 16)	11 (69%)	4 (25%)	1 (6%)	Neither: 8 (50%) Spectacles only: 3 (19%) CLs only: 2 (12%) Both: 3 (19%)	6 (38%) [5 of the 6 wear CL during play]	Of the 8 (50%) with refractive correction, 5 are primarily wearing correction for myopia, 2 for astigmatism, and 1 for hypermetropia	3 (18.8%)	2 of those who have spectacles (for mild myopia and astigmatism correction) do not wear these during play and do not wear contact lenses. In 1 other individual, vision was reduced due to myopia for which no correction was worn (spectacles or contact lenses). Aside from these uncorrected refractive error cases, there were no other residual visual issues that could affect play.
Rugby-league players (*n* = 20)	14 (70%)	3 (15%)	3 (15%)	Neither: 18 (90%) Spectacles only: 2 (10%) CLs only: 0 (0%) Both: 0 (0%)	0 (0%)	Of the 2 players (10%) with refractive correction (spectacles), both are primarily worn for the correction for myopia.	5 (25%)	Neither of the 2 spectacle wearers have contact lenses; hence, no refractive correction is worn by them during play. In 1 other individual, vision was reduced due to myopia for which no correction was worn (spectacles or contact lenses). The remaining 2 visual issues are binocular vision issues. Neither of these were previously diagnosed [i.e. participant unaware]; 1 (convergence insufficiency) is potentially treatable, while the other (ocular motility disorder) is not. The extent to which they would affect vision during play is uncertain.
Near-elite cricketers (*n* = 23)	12 (52.2%)	4 (17.4%)	7 (30.4%)	Neither: 19/23 (82.6%) Spectacles only: 0/23 (0%) CLs only: 0/23 (0%) Both: 4/23 (17.4%)	4 (17.4%) [all 4 wear CL during play]	Of the 4 (17.4%) with refractive correction, 2 are primarily wearing correction for myopia and 2 for hypermetropia.	8 (35%)	6 of the eight cases are refractive in origin (5 where no refractive correction is worn, 1 where only a partial correction is worn). The remaining 2 cases had (a) an ocular disease (macular dystrophy, diagnosed, not treatable, likely to have substantial impact on vision on the field) and (b) a binocular vision issue (convergence insufficiency, potentially treatable, not previously diagnosed, uncertain impact on vision on the field).

*CL* contact lenses

### Statistical Analyses

For each sub-group, we used *z* tests to compare near and distance VA, and stereoacuity against published values from young adults. We also compared performance on each of these tests between the participant sub-groups using *t* tests. For both *z* and *t* tests, we adopted an *α*-criterion of 0.01.

### Ethics, Consent, and Permissions

The study was approved by the ethics committee at the University of Bradford, and the tenets of the Declaration of Helsinki were followed. Written, informed consent was obtained from all participants included in the study.

## Results

### Clinically Measured Level of Visual Function

**Fig. 1:**
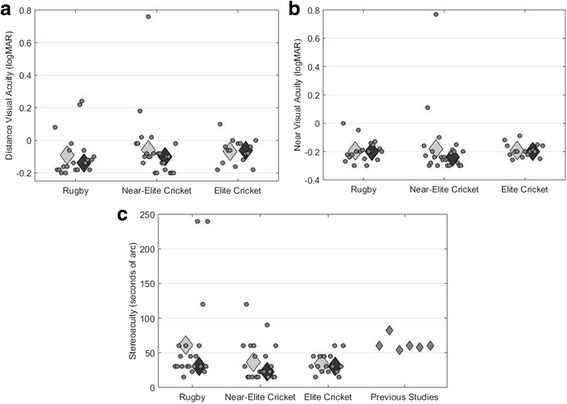
**a** Distance visual acuity (VA) with habitual prescription worn for sports, if any. Circles represent data for individual subjects. These data are jittered on the *x*-axis to enhance legibility. Pale and darker diamond symbols represent the average and median values, respectively (see Table 1). **b** Same as for **a**, except for near VA. **c** Same as **a** except for TNO stereoacuity. Results from previous studies of near stereoacuity in young adults are also shown. From left to right, these data are from [52](median), [52] (average), [51] (average), [53] (average), [54] (average), and [53] (median). There were no differences in distance VA (**a**), near VA (**b**), or stereoacuity (**c**) between the three samples that participated in this study (all *p* > 0.10)

Stereoacuity and VA data are presented in Table 1 and Fig. 1. There were no statistically significant differences in VA (distance or near) between the three subgroups (*p* > 0.01 for all comparisons). In all sub-groups, the average distance VA was ~ 1 line of letters worse than the level which the published literature indicates can be expected in young, optimally corrected, non-sporting adults [49], i.e. those who have undergone an eye examination and are wearing the optical prescription that maximises their visual acuity. However, the deficit we identified in distance VA was only statistically significant for the elite cricketers (Table 1). The modest levels of distance VA mainly reflect the fact that a number of individuals had under- or uncorrected refractive error (see below). This is consistent with the finding that pinhole viewing improved VA by ≥ 1 line in 8.5% of participants (Table 1). Levels of near VA were consistent with those expected in young adults [50] (Table 1, Fig. 1).

Overall, the median near stereoacuity was 30″. Stereoacuity did not differ significantly between the three sub-groups (Table 1, Fig. 1). Stereoacuity in elite cricketers was significantly better (*p* < 0.001) than 50″, which is a consistently reported average value for TNO stereoacuity in young adults [51–55]. However, stereoacuity in the near-elite cricketers (*p* = 0.03) and rugby-league players (*p* = 0.47) was not better than the 50″ criterion. While median stereoacuity was good in all sub-groups (Table 1), three rugby-league players (15%) and two near-elite cricketers (~ 9%) had stereoacuity worse than 60″. These two cricketers were ‘lower-order’ batters, meaning that they are less-able at batting than some of their teammates and that their primary contribution to the team is in the form of bowling and fielding. None of the elite cricketers had stereoacuity worse than 60″.

Colour vision testing suggested normal performance in all participants except two near-elite cricketers. Thus, overall, 4.7% of our males had red/green colour deficiency compared to an expected 8% in European Caucasian males [56], who comprised the overwhelming majority of our males. This discrepancy may be due to our modest sample size, but it is consistent with findings that colour vision deficits are under-represented in high-level cricketers in England [57] and with another study [58] in which fewer first-grade cricketers than expected were found to be colour-vision deficient. None of the female cricketers were found to be colour deficient, and this is consistent with the much lower prevalence of red/green deficiency expected in females compared to males [56].

### Residual Visual Issues

Residual visual issues (i.e. uncorrected, possibly unknown, anomalies of vision/visual system that could impact upon play) were found in 27.1% (16/59) of participants. Amongst the sub-groups, this varied from 18.8 to 35% (Table 2). Of the 16 individuals, most (*n* = 12, 75%) had uncorrected (92%) or under-corrected (8%) refractive error, and the pinhole test revealed improved VA in 5 of these 12 cases. In 6 of the 12 cases, the last eye examination had taken place > 5 years previously or no examination had ever taken place. In 4 of the 12 cases, correction for mild refractive error (up to 1 dioptre of myopia or astigmatism) was worn but not during play.

No cases of undetected ocular pathology existed, but one player (near-elite bowler) had significant, known ocular pathology that impacted on vision during play (Table 3). Three cases of binocular vision anomalies existed (Table 3).

**Table 3:** Questionnaire responses

	Elite cricketers (*n* = 16)	Near-elite cricketers (*n* = 23)	Rugby-league players (*n* = 18)
How would you describe your eyesight?	Excellent: 18.8% Good: 75.0% Moderate: 6.3% Poor: 0%	Excellent: 60.9% Good: 13.0% Moderate: 13.0% Poor: 8.7% No response: 4.3%	Excellent: 38.9% Good: [50.0% Moderate: 5.6% Poor: 5.6%
Do you ever notice problems with your vision when playing?	Y: 31.3% N: 68.7%	Y: 8.7% N: 91.3%	Y: 16.7% N: 83.3%
Do you ever wear sunglasses when playing?	Y: 56.3% N: 31.3% No response: 12.5%	Y: 60.9% N: 30.4% No response: 8.7%	N/A
How frequently do you have your eyes examined?	Every year: 31.3% 1–2 years: 18.8% 2–5 years: 31.3% 5+ years/never: 12.5% No response: 6.3%	Every year: 30.4% 1–2 years: 17.4% 2–5 years: 13.0% 5+ years/never: 34.9% No response: 4.3%	Every year: 27.8% 1–2 years: 11.1% 2–5 years: 5.6% 5+ years/never: 22.2% No response: 33.3%
Did you wear an eye patch as a child?	Y: 0% N: 100%	Y: 0% N: 100%	Y: 5.6% N: 94.4%
Have you have any kind of eye surgery?	Y: 0% N: 100%	Y: 0% N: 95.7% No response: 4.3%	Y: 5.6% (1 case; for detached retina) N: 94.4%
Any injury to your eyes or eye condition that caused you to visit Doctor/Hospital?	Y: 0% N: 100%	Y: 35.3% [not sports-related: 3 × corneal scratch, 1 × conjunctivitis, 1 × macular dystrophy; sports related: 1 × sub-conjunctival haemorrhage caused by ball to face (no lasting damage)]. N: 95.7%	Y: 5.6% (1 case: for detached retina) N: 94.4%
Any difficulty seeing colours?	Y: 0% N: 100%	Y: 4.3% N: 91.4% No response: 4.3%	Y: 0% N: 83.3% No response: 16.7%
Have you ever taken part in an eye/vision training programme designed to improve your sports performance?	Y: 0% N: 100%	Y: 12.9% [in all cases, this was suggested by coach, was ‘cricket specific’ visual training; for 0.5 to 1 h per day over 2–5 months. Deemed ‘useful’ by the player in 2 of 3 cases; ‘not useful’ in other case] N: 87.1%	Y: 0/18 [0%] N: 100%

### Uptake of Eye Care

Sixty-three per cent of all participants had their last eye examination within the past 2 years. However, there were substantial differences between the groups (Table 2); ~ 30% of near-elite cricketers had their last eye examination > 5 years before or had never had one, compared to 15% of rugby-league players and 6% of elite cricketers. Across the full sample, the figure was 18.6% (Table 2). In the questionnaire, only ~ 50% of the cricketers (elite and near-elite) and ~ 40% of the rugby-league players indicated that they regularly undergo eye examinations at least every 2 years (Table 3). Overall, 24.6% (Table 3) said they have eye examinations at ≥ 5 yearly intervals.

### Refractive Correction Worn During Play

17.4% of near-elite cricketers and 38% of elite cricketers reported using refractive correction during play, but none of the rugby-league players did (Table 3). In all except one case, this consisted of contact lenses. 62.2% of cricketers wore (non-corrective) sunglasses in bright playing conditions (Table 3). No participant had refractive surgery.

### Perceived Level of Visual Function

Overall, 84.2% of players rated their vison as ‘good’ or ‘excellent’ (Table 3). Around 20% of the near-elite cricketers rated their vision as only ‘moderate’ or ‘poor’, compared to 11 and 6% of the rugby-league players and elite cricketers, respectively (Table 3). The majority of players (5/8, 62.5%) who rated their vision as ‘moderate’ or ‘poor’ had uncorrected/under-corrected refractive error.

Although over 90% of elite cricketers rated their vision as ‘good’ or ‘excellent’, ~ 30% reported visual difficulties during play, compared with only 16.7% of rugby-league players and 8.7% of near-elite cricketers (Table 3). The discrepancy between the proportion of players with residual visual issues (see above) and the proportion who report visual problems during play for the elite cricketers (18.8 versus 30%, respectively), near-elite-level cricketers (35 versus 8.7%), and rugby-league players (25 versus 16.7%) suggests that perceived level of vision may not be a reliable guide to clinically measured vision and vice-versa (Tables 2 and 3).

### History of Eye Disease, Eye Accidents, and Vision Training

Table 3 contains details of sports-related eye injuries (one rugby-league player), ocular disease (one near-elite cricketer), and vision training (two near-elite cricketers) to improve sports performance.

## Discussion

To our knowledge, there have been no previous studies of eye care and basic (i.e. standard) visual abilities amongst UK-based high-level sports players in the games of cricket or rugby league. This work represents a preliminary attempt to understand whether having optimal and/or superior vision is important for elite-level participation in these sports. Our sample size is modest which reduces the power of our statistical analyses. Having said this, groups of elites are by their very nature small because they represent the best of their sports, e.g. our cricketing elites were, at the time, more or less the entire England’s ladies cricket squad. We did not gather equivalent data in a control group of non-sporting, age-matched individuals. Instead, we compared the results in our sports players with published results from young, optimally corrected, non-sporting adults. Thus, while these comparisons are between samples of broadly similar age, they are not specifically age-matched nor of the same sample size. We believe this was a better approach because published data on young adults are generally of studies involving larger sample sizes, which we believed there was no need to try to replicate.

### Is ‘Excellent’ Vision a Prerequisite for Participation in Elite Cricket/Rugby League?

Our results show that our samples of elite and near-elite sports players do not have basic visual characteristics which could be considered excellent/superior. Aside from the possible exception of near stereoacuity, we did not find superior visual function relative to published values for young, optimally corrected adults [48–55]. The latter finding is at odds with studies of sports people in which the average level of vision (e.g. distance VA) was found to be excellent [5, 59, 60], but it is supported by studies which, like the present study, found that the average level of vision was no better than would be expected compared to the levels found in young adults (e.g. [61, 62]). On a similar note, we are not the first to find a significant proportion of high-performance athletes with either sub-optimal VA or other visual issues [28, 30]. For example, one study [63] reported that 28% of their sample of elite sportspersons had distance VA poorer than + 0.10logMAR using the refractive correction habitually worn for sports.

Since uncorrected/under-corrected refractive error in young adults is more likely to impact on distance vision than on near vision [64], it follows that there might not be an adverse effect on stereoacuity taken at near (40 cm). Indeed, this is what we found, with very good median near stereoacuity in all sub-groups. Previous studies have found better-than-average stereoacuity in elite ball-sports athletes (i.e. baseball) [5, 8, 59], though whether this applies to both distance and near stereoacuity remains unclear [5, 65]. The possible importance of stereoacuity for sports is strengthened by the fact that individuals with naturally occurring, poor stereopsis exhibit poorer catching ability [66] and much reduced learning on a catching task by comparison with those with good stereopsis [67]. Interestingly, with some notable exceptions [21, 22], there are few reports of individuals with poor stereopsis reaching elite levels in sports, though of course this could be the result of children with a binocular disorder not being encouraged to participate in sports. If good or excellent stereoacuity is associated with elite-level sports, it is not clear what the nature of this association is, since disparity processing is temporally slow compared with that of luminance [68]. It is also not clear whether the better stereopsis that we and others [5, 8, 59] have reported in elite sportspeople leads to faster motion-in-depth perception. There appear to be two mechanisms for motion-in-depth perception, one based on sensitivity to retinal disparity and the other based on a comparison of motion signals in the two eyes. The relative importance of these two mechanisms for judging motion-in-depth is still debated [69, 70], but the mechanism comparing motion signals is thought to be independent of the one that uses binocular disparity. Thus, good motion in depth perception is not necessarily dependent on good stereopsis.

### Uptake of Eye Examinations and Prevalence of Visual Disorders

Twenty to twenty-five per cent of our participants reported either never having an eye examination or that their examination interval was ≥ 5 years. This is consistent with a study [63] conducted in the USA between 1992 and 1995 on junior Olympic competitors and in other elites in which 25% had never had an eye examination. Given that we also found that 20–25% of the players had a vision/visual system anomaly in the habitual sports visual status, it is tempting to conclude that simply uncovering and addressing these anomalies through more frequent eye examinations would have a significant impact on the on-field vision of a sizeable proportion of players. This is partly supported by our finding that most residual visual anomalies consisted of uncorrected refractive error in individuals who had not had an eye examination within the past 5 years. However, while some players only became aware of the fact that their vision could be improved as a result of the examination we conducted, this was not the case for all of the players. Notably, four players (25% of those with residual visual anomalies) were aware they required refractive correction, but they chose not to wear it while playing. Also, another four players had anomalies that were non-refractive and which may not be treatable or their impact on vision during play was uncertain. Thus, while greater uptake of eye examinations will improve on-field VA in a selection of players, the proportion likely to benefit is probably considerably smaller than the overall 20–25% in whom we, and others [63], found anomalies.

Beckermann and Hitzeman [63] found that 29% of their sample had visual symptoms. We found that overall 17.5% reported vision difficulties when playing, with the highest proportion of these coming from the elite cricketers. Curiously, this group had the lowest proportion of residual visual anomalies found during clinical examination (Table 2). Discrepancies between perceived and clinically measured deficits in vision may arise because clinical measures do not reflect the visual demands on-the-field or because some reduction in clinically measured visual function can exist without impacting on perceived vision on the field (or both).

### Other ways to Study the Nature of the Relationship between Vision and Elite Sports

The current study has been concerned with understanding whether superior vision is important for elite sports participation, and as such, we examined the basic visual attributes and the visual history of elite/near-elite cricketers and rugby-league players. Other ways to examine the relationship between vision and high-level sports participation/performance include artificially generating a visual problem in visual normals. Mann and colleagues [25, 26] found that low-to-moderate levels of simulated myopia did not affect cricket batting and there are other examples of resistance to defocus in other interceptive tasks [71] and in other sporting tasks (e.g. basketball [23], golf [24]). One interpretation of such findings, and of ours, is that while good or excellent vision may be desirable for elite sports, it may not be essential. Another possibility is that a deficit in basic visual abilities might be overcome by adopting different visual strategies or by gathering the critical information from different cues. An example of the latter is that depth information is recoverable from retinal disparity but may also be available via different means if the disparity signal is reduced (see the ‘Is ‘Excellent’ Vision a Prerequisite for Participation in Elite Cricket/Rugby League?’ section). Investigating whether different visual strategies (e.g. where the eyes are looking, stability of fixation) protect against visual loss due to, for example, uncorrected myopia can be carried out by artificially degrading the retinal image quality in visual normal (e.g. [25, 26]). However, given that it may take time for the visual system to adapt to the deficit it is experiencing, it is likely to prove more informative to conduct such studies in elite-sporting individuals who have been unable to adapt following an acute reduction in vision (e.g. following an accident, [72]) or conversely in those who have reached elite levels in their sports despite habitual, sub-optimal vision. Only a small number of such studies have previously been conducted [21, 22].

To fully understand the nature of the relationship between vision and elite sports, it is important to determine the visual demands during play. Given the dynamic environment of many sports, clinical visual measures gathered in static testing conditions do not reflect the visual demands experienced during play. Hence, researchers have measured performance on non-standard vision tasks (e.g. dynamic VA [4, 12, 36, 73]) and other visual-mediated, cognitive tasks (e.g. [74]). However, different sports can have very different visual demands (e.g. golf versus squash), and even within the same sports, the demands can differ markedly (e.g. bowling versus batting). Identifying the visual demands of different sporting tasks presents a challenge in understanding how vision could limit or enhance the potential for sporting ‘eliteness’. While there have been attempts to study this topic [15, 59, 75, 76], it deserves considerably more research attention. Indeed, it is notable that while there is a growing number of online, web-based training programmes claiming to improve on-field performance (e.g. ‘EyeGym’, ‘Nike Sparq’, ‘Neurotracker’), for the most part, the literature to support their use is scarce [77].

A different, though not incompatible view of the contribution of good vision to eliteness is that visual abilities as measured during standard, clinical testing play a lesser role and that the contribution of vision is to facilitate perceptual-cognitive expertise that emerges via information pickup from anticipatory cues [18]. This is apparent from differences in where and when elite players and elite-sports officials look during play (e.g. [39–43, 78, 79]), in the ability to recall patterns of play (e.g. [80]), and to perform successfully with limited information (e.g. [81]). There is a large volume of literature to support the contribution of perceptual-cognitive expertise to elite sports; specifically, there may not be better vision in elites, just better use of vision (reviewed in [39]). This is compatible with our findings and those of others (e.g. [61–63]) who have concluded that, for the most part, vision as measured in clinical settings is not superior in elite-level sports individuals. However, the importance of optimising vision in elite sportspeople is far from settled because there have been positive outcomes from recent vision training studies, as well as claims that the benefits transfer to better performance on the field [20]. One area of study that is notably missing from the literature concerns intra-individual improvements in performance following the correction of visual anomalies. Such studies need to be carefully designed because of the risk of contamination from placebo effects, but they may provide a more direct answer to the question concerning the importance of optimum vision for elite sporting performance. Other studies that may also prove useful, and which are similarly absent from the literature, would involve comparing visual strategies in those with different habitual levels of basic visual abilities (e.g. due to uncorrected refractive error). Studies of this nature may reveal the extent to which visual deficits might be fully or partially compensated for through the use of different visual strategies.

## Conclusions

With the exception of near stereoacuity (which was superior in our elite cricketers), vision was not superior in our modestly sized sample of high-level, sports players when compared to published values for young adults, and for some measures, it was slightly worse. Moreover, since 20–25% of our sample had non-optimal vision, our findings suggest it is not critical to have the clearest possible vision for high-level sports. In cross-sectional studies like this, it is not possible to say that on-field performance would not be better if vision was improved. Studies of change in performance following the optimization of vision in those with correctable visual anomalies would be useful and would help to better characterise the nature of the relationship between vision and elite sports performance.

## Additional file

Additional file 1: Raw Data for Uploading to Sports Med Open Sept 2017 (XLSX 64 kb)
